# Metastatic Malignant Paraganglioma Presenting as a Neck Mass Treated with Radiolabeled Somatostatin Analog

**DOI:** 10.1155/2021/8856167

**Published:** 2021-06-07

**Authors:** Waqas Jehangir, Alexander Karabachev, Jackie Tsao, Christopher J. Anker, Sree Susmitha Garapati, Janusz K. Kikut, Hibba Tul Rehman

**Affiliations:** ^1^University of Vermont Medical Center, Hematology and Medical Oncology, 89 Beaumont Ave. Burlington, VT 05405-0068, USA; ^2^University of Vermont College of Medicine, Larner College of Medicine, 89 Beaumont Ave. Burlington, VT 05405-0068, USA; ^3^University of Vermont Medical Center, Radiation Oncology, 89 Beaumont Ave. Burlington, VT 05405-0068, USA; ^4^University of Vermont Medical Center, Endocrinology Diabetes & Metabolism, 89 Beaumont Ave. Burlington, VT 05405-0068, USA; ^5^University of Vermont Medical Center, Radiology, 89 Beaumont Ave. Burlington, VT 05405-0068, USA

## Abstract

Paragangliomas are rare neuroendocrine tumors that arise from chromaffin-containing tissue. Surgical resection and/or radiation are used for locoregional disease, and reduction of tumor burden with systemic therapy is reserved for metastatic disease. Iobenguane I-131, somatostatin analog (octreotide), and Sunitinib are noncytotoxic options for treatment, while cyclophosphamide, vincristine, and dacarbazine (CVD) and temozolomide are often used as initial chemotherapy options as studies have shown that they offer some tumor response. However, there are no randomized clinical trials demonstrating prolonged survival with the use of chemotherapeutics in metastatic cases. Investigation of alternative therapies that provide survival benefit is thus necessary. We present a case of a 69-year-old female with metastatic malignant paraganglioma presenting as a left parapharyngeal neck mass, which metastasized after surgery, requiring radiation therapy for bony metastasis who was treated with a radioisotope somatostatin analog for disease progression.

## 1. Introduction

Paragangliomas and pheochromocytomas are rare neuroendocrine tumors that arise from chromaffin-containing tissue. The combined prevalence of these tumors lies roughly between 1 : 6500 and 1 : 2500 with an annual incidence of 500-1600 cases per year in the United States [1]. Sympathetic paragangliomas arise from chromaffin cells along the sympathetic chain in the chest, abdomen, and pelvis, and pheochromocytomas arise from similar cells in the adrenal glands. These two types of tumors are known to synthesize and release catecholamines, which can cause severe hypertension, palpitations, stroke, and possibly death. By contrast, parasympathetic paragangliomas are typically nonfunctional and do not produce symptoms associated with catecholamine hypersecretion. They instead are usually space-occupying lesions derived from parasympathetic nerves in the head, neck, or mediastinum and may cause pain, hoarseness, and dysphagia secondary to a mass effect on surrounding structures [2]. Parasympathetic paragangliomas are often benign but locally invasive. Surgical resection remains the standard therapy as paragangliomas tend to respond poorly to chemotherapy and radiation [3].

Though typically benign, parasympathetic paragangliomas can become malignant and metastasize. Because spread is rare, there is currently no standard treatment for metastatic malignant paragangliomas. Cyclophosphamide, vincristine, and dacarbazine (CVD) are often used as initial chemotherapy as studies have shown that they offer some tumor response [4]. However, there are no randomized clinical trials demonstrating prolonged survival with the use of chemotherapeutics in metastatic cases [5]. Investigation of alternative therapies that provide survival benefit is thus necessary. We present the case of a 69-year-old female with metastatic malignant paraganglioma who was treated with a somatostatin analog for widespread disease refractory to surgery, radiation, and targeted therapy.

## 2. Case Presentation

**Figure 1:**
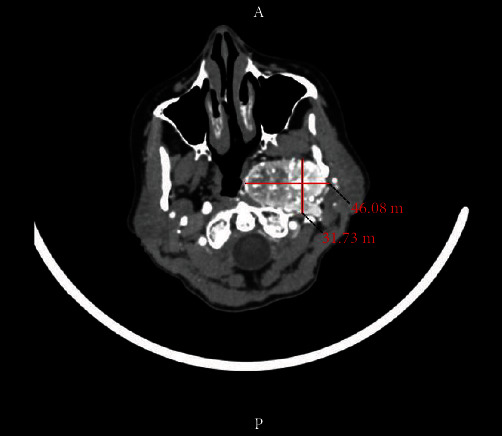
CT neck showing encasement and marked narrowing of the left cervical internal carotid artery.

A 69-year-old female with no smoking history or family history of paragangliomas or other neuroendocrine tumors initially noted discomfort in her left neck in 2007 which was thought to be related to a cervical disk pathology; however, by March 2010, her symptoms had persisted and then she had laryngitis for about 5 weeks. She was seen by her doctor who could not hear her left carotid, and she was also noted to have a grayish appearance. A left tonsillar lesion was noted, which was felt to be secondary to medial disposition of the tonsil from some extrinsic compression. A mass was noted at the left angle of the jaw in level 2. She was prescribed antibiotics; however, they did not improve her symptoms and vascular surgery was consulted. A CT angiogram of the neck was performed in 2010. Contiguous axial CT images were obtained of the neck using 80 mL of Isovue-370 IV contrast followed by 60 mL of normal saline infused at 6 mL/seconds. A 4.6 × 3.2 × 5.5 cm heterogenous attenuation, hypervascular mass appearing to arise from the left retrostyloid parapharyngeal space was most consistent with a paraganglioma, likely globus vagale. Encasement and marked narrowing of the left cervical internal carotid artery were noted (Figure 1).

MRI was performed which further demonstrated a large left cervical mass causing markedly diminished signal in the cervical left internal carotid artery likely representing diminished flow due to compression by the mass. Prior to surgery, she was put on alpha and beta blockade. She underwent embolization as well as excision of the tumor by a combination of transcervical and transoral approaches with lip and mandibular split. Her tumor was encroaching the left jugular foramen with a persistent tumor evident extending up to the jugular foramen at the termination of the procedure, despite intentional sacrifice of the cranial nerves 9, 10, and 12, given their relationship to the tumor. There was a small portion of the residual tumor that could not be resected at the superior portion of the jugular foramen. The pathology showed an extra-adrenal pheochromocytoma (paraganglioma) from the left carotid space, superior to the carotid bifurcation. Immunohistochemical staining was positive for chromogranin, synaptophysin, and S-100 and was faint for glial fibrillary acidic protein (GFAP). It measured 4.3 × 3.8 × 2.5 cm positive lymphovascular invasion, the mitotic rate is 1/10 high-power field, and necrosis was present, likely due to embolization. The margins were positive. She was on surveillance after surgery.

**Figure 2:**
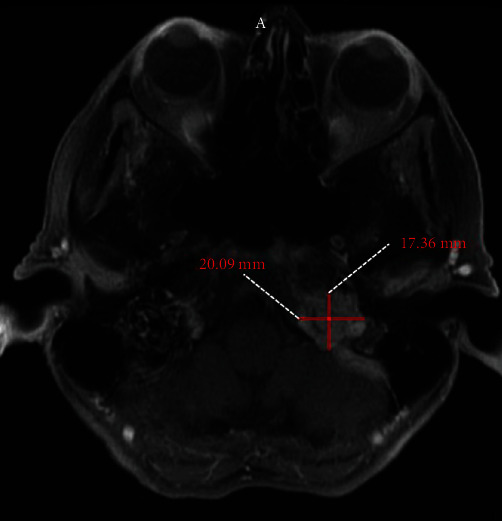
MRI of the neck with and without contrast showing a mass lesion centered within the left jugular foramen, appearing mildly increased in size. The size was 20 mm in maximal dimension as opposed to 17 mm compared to previous imaging 6 months prior.

Since her surgery, sequential MRI scans of the brain showed a slowly progressive recurrence. Surgery was not recommended due to potential morbidity. In 2015, she underwent stereotactic radiosurgery at a dose of 15 Gy to the left paraganglioma. In 2016, MRI of the neck with and without contrast revealed a mass lesion centered within the left jugular foramen, appearing mildly increased in size with an unchanged mild mass effect upon the adjacent left cerebellar hemisphere. The size was 20 mm in maximal dimension as opposed to 17 mm compared to previous imaging 6 months prior (Figure 2). A CT scan of the chest showed scattered noncalcified pulmonary nodules. The largest was noted to be in the right middle lobe, demonstrating no discernible growth based on the volumetric analysis compared to previous imaging. Her PET/CT revealed FDG uptake in the right middle lobe pulmonary nodule. She was also found to have FDG uptake within bony lesions involving T3, T7, L3, and the T1 transverse process consistent with metastasis. FDG uptake was noted within the biopsy-proven left jugular foramen paraganglioma. She underwent CT-guided FNA of a 6 mm right middle lobe lesion which revealed neoplastic, low-grade tumor, favoring a neuroectodermal tumor, and after discussion in MDC, it was concluded that her paraganglioma disease was likely metastatic to the lung and spine. She underwent stereotactic ablative radiation therapy to the T7 and T3 paraganglioma metastasis to the spine to a dose of 1600 cGy.

**Figure 3:**
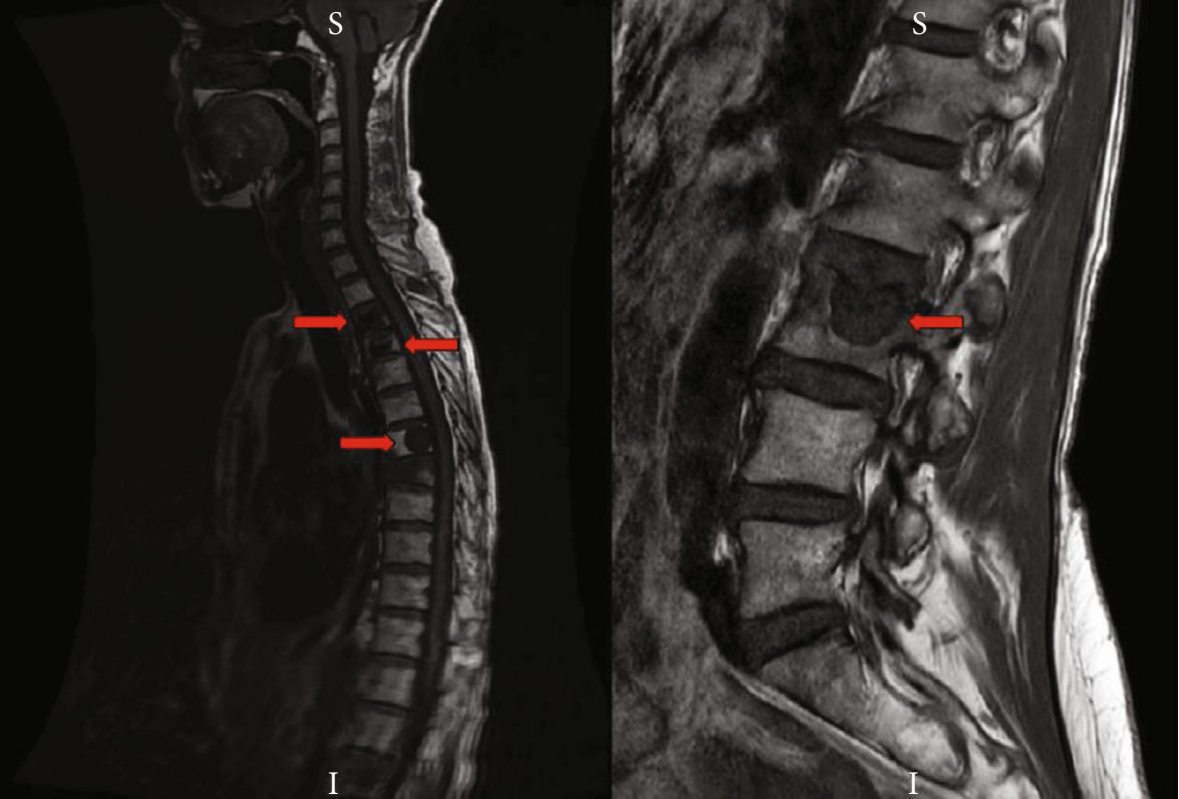
MRI of the thoracic and lumbar spine showing multiple osseous lesions.

In 2017, she had a T4 vertebral body biopsy and kyphoplasty for her back pain secondary to metastasis. In the fall of 2017, she was found to have a compression fracture and underwent T3 vertebroplasty with improvement in pain. Her MRI of the thoracic and lumbar spine showed multiple osseous lesions (Figure 3). It also showed metastatic disease including the stable lung nodule which was biopsied and mild degenerative disc disease. In January 2018, CT of the chest abdomen pelvis showed stable disease (T3 and T7). CT of the neck showed stable disease in the left jugular foramen. In the spring of 2018, she was diagnosed with a T8 compression fracture and underwent T8 vertebroplasty. She was started on monthly denosumab. In the fall of 2018, CT chest showed stable lung nodules and CT abdomen pelvis showed mixed sclerotic and lytic lesions at L1 and L3 with increased compression of the left side of the superior endplate of L3 since the prior CT. MRI showed that the metastatic lesions in the L-spine were slightly larger without any new lesions. Soft tissue mass in the left jugular foramen appears to be stable without any cervical lymphadenopathy; however, there was a concern of C3 metastatic lesion and metastatic lesions in the thoracic spine that also increased in size. She was referred for genetic testing, and the results came back positive for a mutation in SDH-C, c.397C>T (p.Arg133∗), which is associated with autosomal dominant hereditary paraganglioma-pheochromocytoma syndromes, gastrointestinal stromal tumors, and renal cell carcinoma [6]. This sequence results in a premature translational stop signal in the penultimate exon of the SDHC mRNA at codon 133 which is expected to delete the last 37 amino acids of the SDHC protein. This variant has been reported in several individuals with paragangliomas [7–10].

**Figure 4:**
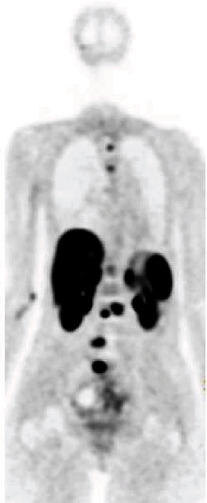
PET/CT GA-68 dotatate scan showing multiple scattered metastatic lesions to the spine with subtle increase in size and conspicuity of a few sacral and lumbar lesions.

**Figure 5:**
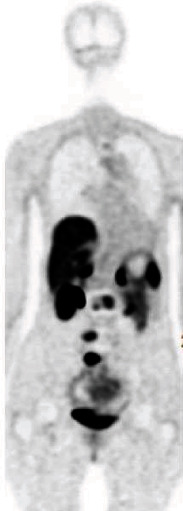
PET/CT GA-68 dotatate scan showing a modest response to therapy with no new lesions identified.

Her case was discussed with local and national experts, and it was recommended to get a gallium 68 dotatate PET scan (as MIBG is not helpful after the lesions have been radiated). She underwent a PET/CT GA-68 dotatate scan in the spring of 2019 which revealed multiple scattered metastatic lesions to the spine with subtle increase in size and conspicuity of a few sacral and lumbar lesions (Figure 4). She was started on lutetium 177 dotatate (Lu 177), a radiolabeled somatostatin analog, in April 2019 and received a half dose in the first cycle due to concerns around catecholamine storm. She had borderline elevated 24-hour urine metanephrine and mildly elevated norepinephrine and dopamine level on plasma catecholamine fractionation and was treated with alpha and beta blockade prior to Lu-177 treatment. Other labs performed prior to treatment include a basic metabolic panel which was all within normal limits, liver function tests which were within normal limits, a complete blood count with a hemoglobin of 10.2 gm/dl, normal platelet count of 368 K/cmm, and normal white blood cell count of 4.82 K/cmm. Her folate was elevated at 24 ng/ml. She tolerated the first cycle and received the full second dose in June 2019. A follow-up PET/CT GA-68 dotatate scan in December 2019 showed a modest response to therapy with no new lesions identified while previously these lesions had been progressively getting worse (Figure 5). During the most recent oncology follow-up visit in February 2020, the patient reports some continued fatigue that is beginning to improve.

## 3. Discussion

Paragangliomas have varied clinical presentations depending on tumor location and size, catecholamine secretory function, and extent of spread [11]. Biochemical testing is encouraged even for patients who do not exhibit symptoms associated with catecholamine hypersecretion. Catecholamines are metabolized into metanephrines (normetanephrine, metanephrine) in chromaffin cells. In tumors, this process occurs independently of catecholamine release. Diagnosis involves 24 h urine collection to assess for the presence of fractionated metanephrine and catecholamines. Several studies have demonstrated that measurement of urinary metanephrines allows for greater diagnostic sensitivity than measurement of serum metanephrines [1]. Histological studies are then performed for definitive diagnosis [12].

A diagnosis of malignant paraganglioma requires the presence of metastasis [1]. Patients with metastatic involvement have an estimated 5-year survival of <50% [5]. Malignant disease is difficult to treat because metastatic paragangliomas are often refractory to chemotherapy and radiation and recurrence is common. A lifelong follow-up is strongly encouraged. Iobenguane I-131, somatostatin analogs (e.g., octreotide), and Sunitinib are targeted options for treatment, while cyclophosphamide, vincristine, and dacarbazine (CVD) and temozolomide are often used as initial chemotherapy options as studies have shown that they offer some tumor response [13–18]. Patients with rapid disease progression are usually treated with chemotherapy or temozolomide, while patients with slow to moderate disease progression are treated with radiolabeled therapies. CVD is the recommended option for rapidly progressive metastatic disease, and the specific regimen includes a 21- to 28-day cycle of cyclophosphamide 750 mg/m^2^ and vincristine 1.4 mg/m^2^ on day 1 and dacarbazine 600 mg/m^2^ on day 1 and 2 [4]. For the SDH-B mutant, CVD is the initial treatment choice while metronomic temozolomide is recommended for patients that do not tolerate the alkylating agents. Another established therapy for metastatic paraganglioma is MIBG therapy and is the preferential therapy for slow growing MIBG-positive metastatic disease [19].

The combination of CVD as an initial chemotherapy regimen was shown to improve symptoms for patients as well as have a high response rate with a median survival of 39 months after initiating treatment [4]. However, a study in 2008 by Huang et al. evaluated eighteen patients 22 years after treatment with CVD for malignant paraganglioma. They reported that the overall survival of patients who had a positive response to CVD did not differ from patients whose disease remained stable or even progressed after treatment. An improvement in symptoms was noted when tumor shrinkage occurred concluding that CVD may be an option to manage symptoms in patients and also for patients where tumor shrinkage may make surgical resection an option [4, 5]. Currently, no chemotherapy regimen has been shown to prolong survival for metastatic paraganglioma.

Radionuclide therapy may be an option for symptom palliation and tumor regression or stabilization. The effectiveness of this treatment is largely dependent on whether the tumor takes up MIBG or somatostatin analogs that the beta-emitting isotopes are coupled to. This is evaluated using Iobenguane I-131 scintigraphy for MIBG or PET imaging using gallium-68-labeled somatostatin analogs [20, 21]. Our patient had positive uptake with the PET/CT GA-68 dotatate scan and was therefore started on lutetium 177 dotatate (Lu 177), a radiolabeled somatostatin analog. It is likely that the increased accuracy of the PET/CT GA-68 dotatate scan is due to the SDH-C deficient status of the patient. In addition to paragangliomas, gastrointestinal stromal tumors, and renal cell carcinoma, SDH mutations have been shown to be related to pituitary tumors [6–10, 22].

Studies have revealed conflicting results for the use of radiolabeled somatostatin analogs for metastatic pheochromocytoma/paraganglioma. Forrer et al. in 2008 showed that radiolabeled somatostatin analog [DOTA-Tyr(3)]-octreotide (DOTATOC) was effective for patients with receptor-positive paraganglioma. They analyzed 28 patients with surgically incurable disease. The treatment was tolerated well, and of these patients, two had partial remissions, five had minor responses and thirteen had stable disease with a mean follow-up of 19 months. From these results, the authors concluded that DOTATOC may be an effective treatment for paraganglioma but is less effective when compared to its response with gastroenteropancreatic neuroendocrine tumors [23].

In 2018, lutetium Lu-177 dotatate (177Lu-dotatate) was approved by the US FDA for the treatment of gastroenteropancreatic neuroendocrine tumors that express somatostatin receptors. This approval did not include paraganglioma/pheochromocytoma. Our case highlights its use in the setting of somatostatin receptor-positive paraganglioma in hopes of appropriate future application of somatostatin analogs for the treatment of malignant paragangliomas. An ongoing trial by NCI is evaluating the role of Lu-177 for inoperable pheochromocytoma and paraganglioma and is expected to be completed in 2023 (NCT03206060).

## Data Availability

All data are available in the manuscript, in the references, or in the ClinicalTrials.gov website.
